# Increased serum IL-36γ levels are associated with disease severity in myasthenia gravis patients

**DOI:** 10.1186/s12883-020-01885-z

**Published:** 2020-08-19

**Authors:** Qiu-Xia Zhang, Yue Li, Shu-Min Jiang, Lin-Jie Zhang, Ming Yi, Jing Wang, Yuan Qi, Li Yang, Chun-Sheng Yang

**Affiliations:** Department of Neurology and Tianjin Neurological Institute, Tianjin Medical University General Hospital, 154 Anshan Road, Heping District, Tianjin, 300052 China

**Keywords:** Biomarkers, Interleukin 36, Myasthenia gravis, Human

## Abstract

**Background:**

Interleukin 36 (IL-36), as a gradually recognized cytokine, is involved in the occurrence and evolution of autoimmune diseases. Nevertheless, the relationship between myasthenia gravis (MG) and IL-36 is rarely reported.

**Methods:**

We evaluated the serum levels of IL-36 (IL-36α, IL-36β and IL-36γ) by enzyme-linked immunosorbent assay (ELISA). Further, clinical parameters in 97 MG patients and 49 healthy controls (HCs) were carefully measured.

**Results:**

Serum IL-36γ levels were significantly elevated in the MG patients compared with the HCs (*p* < 0.0001). Compared to those in remission, patients in the acute phase exhibited higher levels of IL-36α and IL-36γ (*p* = 0.038 and *p* = 0.011, respectively). Furthermore, patients with generalized MG (GMG) exhibited markedly higher serum IL-36γ levels than those with ocular MG (OMG) (*p* = 0.003).

**Conclusions:**

The serum levels of IL-36γ in patients with MG were increased and positively correlated with disease severity and may thus have potential as a serological MG marker.

## Background

Myasthenia gravis (MG) is an antibody-mediated inflammatory disease in which antibodies mainly destroy acetylcholine receptors (AChRs) at the junction between the nerve and muscle.

MG patients exhibit many other autoimmune antibodies [1–3], such as anti-lipoprotein-related protein 4 (LRP4), anti-titin, anti-ryanodine, anti-agrin, anti-cortain antibodies and anti-muscle-specific tyrosine kinase (MuSK), in addition to anti-AChR antibodies. Moreover, cytokines can regulate immune responses and contribute to MG pathogenesis, which impairs the transmission of neuromuscular junctions [4, 5].

Interleukin 36 (IL-36), a new member of the IL-1 family, binds to the IL-36 receptor (IL-36R), which further activates both dendritic cells and CD4+ T lymphocytes, exerting a proinflammatory effect [6].

The latest evidence shows that IL-36 is involved in many autoimmune diseases, including systemic lupus erythematosus (SLE), atopic dermatitis and multiple sclerosis [7–12]. At present, the serum levels of IL-36 in MG patients are rarely reported. In our current research, we evaluated serum IL-36 levels in MG patients and their correlation with clinical characteristics.

## Methods

### Patients

We enrolled 97 MG patients who were treated at the Department of Neurology, Tianjin Medical University General Hospital between January 2016 and September 2019. At the same time, 49 healthy controls (HCs), were recruited for the study. The patients with MG were diagnosed according to the typical clinical manifestations of weakness in voluntary muscles and those patients meet at least one of the following three criteria [13]: (1) positive anti-AChR antibody detection in serum; (2) repetitive motor nerve stimulation (RNS) decrement of 10% or greater; and (3) a positive response to intramuscular neostigmine. The exclusion criteria were as follows: (1) below 18 years of age and (2) history of previous inflammatory, neoplastic or other autoimmune disease. HCs were enrolled from the Health Care Center of our hospital. And those accompanied by autoimmune diseases, infectious diseases, heart diseases, lung diseases, kidney diseases were excluded.

The study was approved by the Ethics Committee of Tianjin Medical University General Hospital, and all the participants provided written consent.

### Sample collection

Clinical data, including sex, age, anti-AChR antibody status, thymoma status, MG Foundation of America (MGFA) classification and MG-ADL score, were acquired from medical records in the electronic system of our hospital. In the acute phase, clinical evaluation and sample collection of 97 patients with MG was completed before treatment (intravenous immunoglobulin, plasma exchange or high-dose corticosteroids for 3–5 consecutive days). In total, 30 patients with MG were randomly selected, and specimens collected during remission were obtained. Simultaneously, general demographic data and fasting venous blood samples were collected from 49 HCs at our hospital. The samples were stored at − 80 °C.

### Serum IL-36 levels

The serum levels of IL-36α, IL-36β and IL-36γ were measured using human IL-36α, IL-36β and IL-36γ enzyme-linked immunosorbent assay (ELISA) kits (R&D Systems), respectively. All the steps were performed strictly in accordance with the kit instructions.

### Statistical analyses

Statistical Program for Social Sciences (SPSS 22.0) was used for statistical analysis, and graphs were created using GraphPad Prism 6.01. IL-36α, IL-36β and IL-36γ, as continuous numerical variables, were compared between two groups using t-tests or the Mann-Whitney U test; gender, as qualitative data, was analyzed using the Fisher’s exact test; and correlation analysis between serum IL-36 levels and MG-ADL scores was performed by Pearson correlation coefficient. *p* < 0.05 was considered statistically significant.

## Results

### Clinical characteristics

The clinical features of the 97 MG patients and 49 HCs are shown as follows: among these individuals, the numbers of women were 44 (45.36%) and 30 (61.22%), and the ages at sampling were 57.30 ± 1.59 years and 56.84 ± 2.72 years, respectively. Similar to the findings of previous studies [14, 15], the results showed that anti-AChR antibodies were detectable in serum samples from 79 (81.44%) of the MG patients, and there were 25 (25.77%) MG patients with thymoma in the current study. The average MG-ADL score was 4.46 ± 3.29, and the distribution of the MG patients in the different MGFA classification categories (I: II: III: IV: V) was 40: 29: 19: 4: 5. In this study, we defined mild MG patients as those who were classified as I or II by MGFA, and the rest of the patients were considered to be severe MG patients.

### Serum IL-36 levels

**Fig. 1:**
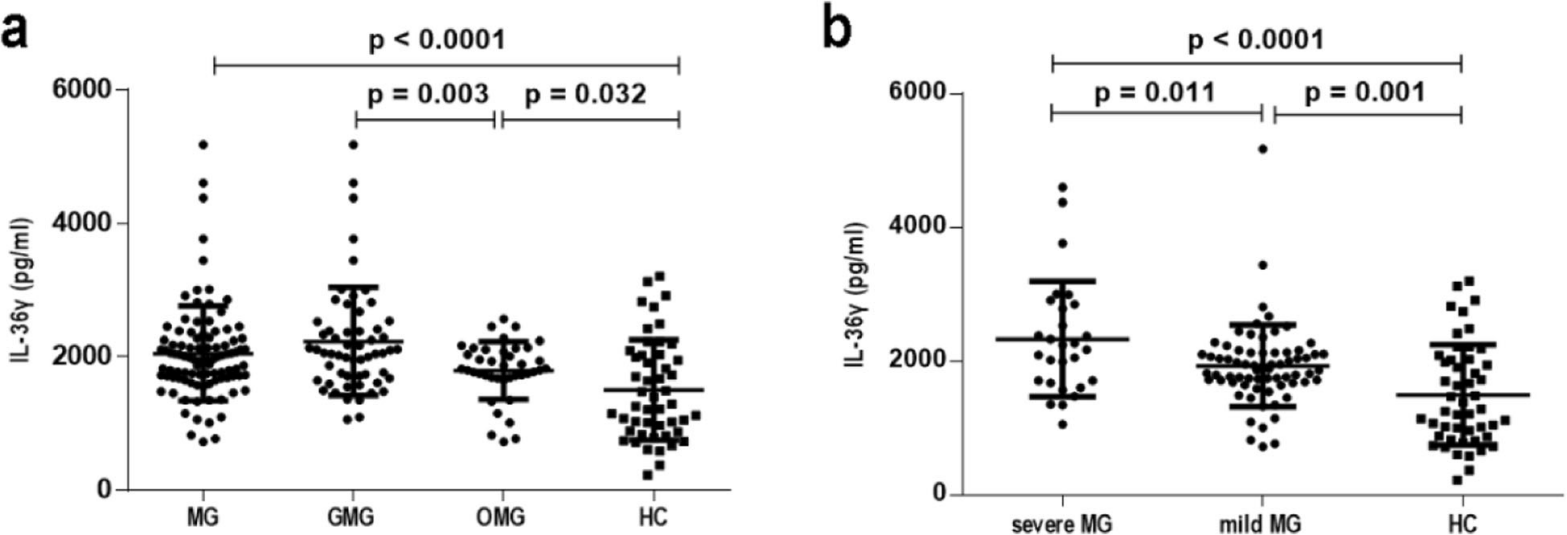
Serum IL-36γ levels in myasthenia gravis (MG) patients and healthy controls (HCs). **a** Comparison of serum IL-36γ levels among MG patients, generalized MG (GMG) patients, ocular MG (OMG) patients and HCs. **b** Comparison of serum IL-36γ levels among severe MG patients, mild MG patients and HCs

**Fig. 2:**
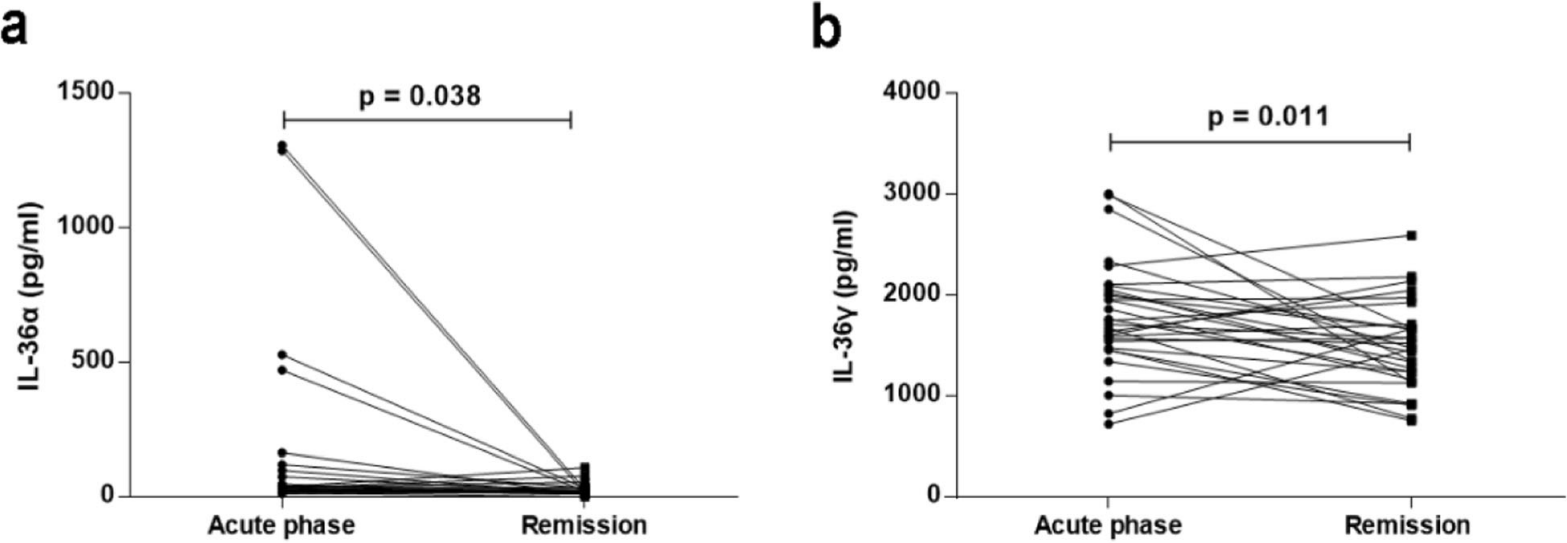
Serum IL-36α and IL-36γ levels in myasthenia gravis (MG) in the acute and remission phases. **a** Comparison of serum IL-36α levels between MG patients in the acute and remission phases. **b** Comparison of serum IL-36γ levels between MG patients in the acute and remission phases

Markedly higher serum IL-36γ levels were found in the MG patients than in the HCs (*p* < 0.0001, Fig. 1a), but there were no significant differences in the serum IL-36α and IL-36β levels between the two groups (*p* = 0.325 and *p* = 0.133, respectively). When comparing different subtypes of MG, we observed that the serum IL-36γ levels in ocular MG (OMG) patients were significantly lower than those in generalized MG (GMG) patients (*p* = 0.003, Fig. 1a) but significantly higher than those in the HCs (*p* = 0.032, Fig. 1a). Furthermore, as illustrated in Fig. 1b, the serum IL-36γ levels in mild MG patients were significantly higher than HCs (*p* = 0.001), but lower than severe MG patients (*p* = 0.011). However, no significant differences in serum IL-36α or IL-36β levels were found between the OMG and GMG patients (*p* = 0.942 and 0.630, respectively). Serum IL-36α and IL-36β levels were equivalent between the mild and severe MG patients (*p* = 0.478 and 0.989, respectively). Furthermore, the serum IL-36α, IL-36β or IL-36γ levels did not differ between anti-AChR antibody-positive and anti-AChR antibody-negative MG patients (*p* = 0.217, 0.393 and 0.829, respectively). Serum IL-36α, IL-36β and IL-36γ levels also showed no significant differences between MG patients with thymoma and those without thymoma (*p* = 0.234, 0.167 and 0.911, respectively). Moreover, the MG patients in remission period had decreased serum IL-36α and IL-36γ levels compared to the acute phase (*p* = 0.038 and 0.011, respectively, Fig. 2). However, serum IL-36β levels did not significantly differ in MG patients between the acute and remission phases (*p* = 0.918).

Moreover, we analyzed the correlation between serum IL-36 levels and MG-ADL score to discover possible intrinsic connections. The Pearson correlation results showed a weak relationship to be considered of any relevant value between the serum IL-36γ levels and the MG-ADL score in MG patients (r = 0.215, *p* = 0.035). Furthermore, serum IL-36α or IL-36β levels and the MG-ADL score were not correlated (r = 0.035, *p* = 0.973; r = 0.078, *p* = 0.451).

## Discussion

Numerous studies have indicated that IL-36 is involved in the immune inflammatory response and may play critical roles in autoimmune diseases. However, the specific mechanism of IL-36 in MG is still rarely reported. In this study, we clarified that serum IL-36γ levels were elevated in MG patients compared with HCs. The clinical manifestations of patients with GMG are more serious, which can manifest as weakness of the limbs, trunk, throat and respiratory muscles. There were 57 patients with GMG, including 28 severe MG patients in current study, and comparisons of subtypes of MG revealed that GMG and severe MG exhibited elevated levels of IL-36γ. Moreover, our study demonstrated that serum IL-36γ levels were decreased during remission. In a few patients with MG, the serum levels of IL-36γ did not decrease during the remission period, but even increased. Which may be due to individual differences, but the overall was still reduced.

As a factor promoting the inflammatory response, IL-36 is involved in the inflammatory process and accelerates the downstream inflammatory response, which has been illustrated in many diseases. A clinical trial of 72 systemic lupus erythematosus (SLE) patients and 63 HCs showed that serum IL-36α and IL-36γ levels were increased in SLE patients and related to systemic lupus erythematosus disease activity index (SLEDAI) scores, which suggest that IL-36 is associated with SLE disease activity [14, 15]. Moreover, another study showed that IL-36α and IL-36γ were expressed to promote the development of inflammation by inducing the production of chemical factors in inflammatory bowel disease [10]. Similar to the above diseases, MG is also an antibody-mediated, cellular immunity-dependent, complement-associated, cytokine-mediated autoimmune disease. Therefore, IL-36 is likely to play a similar role in MG.

Although many studies have explored the pathogenesis of IL-36, we know very little about the specific mechanisms of IL-36, and thus, IL-36 remains to be further explored. The latest demonstrates that IL-36 is secreted by not only peripheral blood cells such as macrophages, DCs and T cells but also keratinocytes and mucosal epithelial cells [10]. Moreover, studies by Towne et al. [16] have shown that IL-36 performs its role through binding to IL-1 receptor-related protein 2 (IL-1Rrp2), which activates the pathway leading to NF-κB. IL-36 has also been shown to activate the MAPK pathway, which is similar to the signaling pathways of IL-1β and IL-18, in NCI/ADR-RES cells. On the one hand, IL-36 stimulates DCs and induces CD4+ T cells to differentiate into Th1 cells [6, 17]. Th1 cells produce IL-2 and IFN-γ, perform helper functions to support particular immunoglobulin classes and subclasses. An imbalance among Th1, Th2 and Th17 cells will participate in the pathological process and aggravate the progression of MG diseases [18–21], so IL-36 may also be involved in the pathogenesis of MG through these cells. On the other hand, IL-36 can greatly promote the secretion of inflammatory factors, including IL-1, IL-6, IL-8, IL-12, IL-18, IL-23, IFN-γ, TNF-α and granulocyte-macrophage colony-stimulating factor, by DCs, macrophages and keratinocytes [16, 22, 23]. Emerging data have shown that these factors also contribute to the pathological process of MG [24–29]; for example, in a study of AChR-immunized IL-6^−/−^ mice, decreases in the anti-AChR antibody titer and C3 levels, and a relatively low proportion of the mice developed experimental autoimmune MG [29]. Consistent with this study, another study found that anti-IL-6 antibodies could inhibit experimental autoimmune MG by reducing the anti-AChR antibody titer and the number of B cells and downregulating the expression of Th17-related genes [30]. Similarly, other studies have clarified that the application of anti-TNF-α antibodies can delay disease onset and lead to disease presentation as only mild muscle weakness [24], and we thus hypothesize that serum IL-36 is involved in the pathogenesis of MG through the interaction of these factors. In conclusion, IL-36 is likely to participate in the pathological process of MG through the abovementioned immune cells and cytokines.

The limitations of this study need to be noted. First, because this study is a cross-sectional study, it is prone to bias. Second, this study enrolled 97 MG patients and 49 HCs in a single-center, small-sample study. Third, though serum samples from patients were collected before treatment, some patients with MG were on oral steroids, and its effect on results is unknown. In the future, we will continue to expand the sample size to discover additional valuable information about IL-36 and MG.

## Conclusions

Our results indicate that IL-36γ is positively correlated with the severity of MG. Additionally, IL-36γ may be involved in the immunopathological process of MG, suggesting that it may be a potential immune marker for MG.

## Data Availability

The datasets used and/or analysed during the current study are available from the corresponding author on reasonable request.

## Abbreviations

IL-36: Interleukin 36
MG: Myasthenia gravis
ELISA: Enzyme-linked immunosorbent assay
HCs: Healthy controls
AChRs: Acetylcholine receptors
MuSK: Muscle-specific tyrosine kinase
GMG: Generalized MG
OMG: Ocular MG
MG-ADL: MG activities of daily living
SLE: Systemic lupus erythematosus
RNS: Repetitive motor nerve stimulation
MGFA: MG Foundation of America
EAMG: Experimental autoimmune MG
